# Reproducibility of Hospital Rankings Based on Centers for Medicare & Medicaid Services Hospital Compare Measures as a Function of Measure Reliability

**DOI:** 10.1001/jamanetworkopen.2021.37647

**Published:** 2021-12-07

**Authors:** Laurent G. Glance, David R. Nerenz, Karen E. Joynt Maddox, Bruce L. Hall, Andrew W. Dick

**Affiliations:** 1 Department of Anesthesiology and Perioperative Medicine, University of Rochester School of Medicine, Rochester, New York; 2 Department of Public Health Sciences, University of Rochester School of Medicine, Rochester, New York; 3 RAND Health, RAND, Boston, Massachusetts; 4 Center for Health Policy and Health Services Research, Henry Ford Health System, Detroit, Michigan; 5 Department of Medicine, Washington University in St Louis, St Louis, Missouri; 6 Center for Health Economics and Policy at the Institute for Public Health, Washington University in St Louis, St Louis, Missouri; 7 Department of Surgery, Washington University in St Louis, St Louis, Missouri; 8 Olin Business School, Washington University in St Louis, St Louis, Missouri; 9 Division of Research and Optimal Patient Care, American College of Surgeons, Chicago, Illinois

## Abstract

**Importance:**

Unreliable performance measures can mask poor-quality care and distort financial incentives in value-based purchasing.

**Objective:**

To examine the association between test-retest reliability and the reproducibility of hospital rankings.

**Design, Setting, and Participants:**

In a cross-sectional design, Centers for Medicare & Medicaid Services Hospital Compare data were analyzed for the 2017 (based on 2014-2017 data) and 2018 (based on 2015-2018 data) reporting periods. The study was conducted from December 13, 2020, to September 30, 2021. This analysis was based on 28 measures, including mortality (acute myocardial infarction, congestive heart failure, pneumonia, and coronary artery bypass grafting), readmissions (acute myocardial infarction, congestive heart failure, pneumonia, and coronary artery bypass grafting), and surgical complications (postoperative acute kidney failure, postoperative respiratory failure, postoperative sepsis, and failure to rescue).

**Exposures:**

Measure reliability based on test-retest reliability testing.

**Main Outcomes and Measures:**

The reproducibility of hospital rankings was quantified by calculating the reclassification rate across the 2017 and 2018 reporting periods after categorizing the hospitals into terciles, quartiles, deciles, and statistical outliers. Linear regression analysis was used to examine the association between the reclassification rate and the intraclass correlation coefficient for each of the classification systems.

**Results:**

The analytic cohort consisted of 28 measures from 4452 hospitals with a median of 2927 (IQR, 2378-3160) hospitals contributing data for each measure. The hospitals participating in the Inpatient Prospective Payment System (n = 3195) had a median bed size of 141 (IQR, 69-261), average daily census of 70 (IQR, 24-155) patients, and a median disproportionate share hospital percentage of 38.2% (IQR, 18.7%-36.6%). The median intraclass correlation coefficient was 0.78 (IQR, 0.72-0.81), ranging between 0.50 and 0.85. The median reclassification rate was 70% (IQR, 62%-71%) when hospitals were ranked by deciles, 43% (IQR, 39%-45%) when ranked by quartiles, 34% (IQR, 31%-36%) when ranked by terciles, and 3.8% (IQR, 2.0%-6.2%) when ranked by outlier status. Increases in measure reliability were not associated with decreases in the reclassification rate. Each 0.1-point increase in the intraclass correlation coefficient was associated with a 6.80 (95% CI, 2.28-11.30; *P* = .005) percentage-point increase in the reclassification rate when hospitals were ranked into performance deciles, 4.15 (95% CI, 1.16-7.14; *P* = .008) when ranked into performance quartiles, 1.47 (95% CI, 1.84, 4.77; *P* = .37) when ranked into performance terciles, and 3.70 (95% CI, 1.30-6.09; *P* = .004) when ranked by outlier status.

**Conclusions and Relevance:**

In this study, more reliable measures were not associated with lower rates of reclassifying hospitals using test-retest reliability testing. These findings suggest that measure reliability should not be assessed with test-retest reliability testing.

## Key Points

**Question:**

To what extent does better measure reliability, quantified using test-retest reliability testing, lead to more reproducible hospital rankings?

**Findings:**

In this cross-sectional study of 28 measures from 4452 hospitals, increases in measure reliability were not associated with improvements in the reproducibility of hospital rankings. The reproducibility of hospital rankings improved with more reliable measures when hospitals with fewer than 500 case were excluded.

**Meaning:**

The findings of this study suggest that test-retest reliability testing should not be used to assess the reliability of performance measures.

## Teaser

This cross-sectional study examines the use of Centers for Medicare & Medicaid Services Hospital Compare data to measure the reliability of hospital rankings.

## Introduction

The Affordable Care Act and the Medicare Access and Children's Health Insurance Program Reauthorization Act were intended to expand health insurance coverage, improve health care quality, and control the growth of health care spending. These landmark legislations led to the creation of the Medicare Shared Savings Program,^1^ Hospital Readmission Reduction Program,^2^ Hospital Value-Based Purchasing,^3^ Bundled Payment for Care Improvement,^4^ and Merit-based Incentive Payment System.^5^ These programs all aim to constrain the growth of health care spending and improve patient outcomes by shifting reimbursements to value-based payments. Because quality measurement is central to value-based purchasing, the scientific acceptability of the performance measures used in these programs is of paramount importance. Scientifically invalid performance measures will distort the financial incentives in value-based purchasing and may fail to promote higher-value care.

For performance measurement to be credible, the performance of hospitals must be accurately measured to distinguish higher-performance hospitals from lower-performance hospitals. Measure reliability is integral to the measure evaluation process used by the National Quality Forum (NQF) to certify whether measures can be used in Medicare value-based purchasing programs.^6^ A hospital performance measure is considered reliable if repeated measurements of the same hospital's performance agree with one another. In other words, a performance measure is reliable if the results are reproducible. Reliability is quantified on a 0 to 1 scale using either the signal-to-noise ratio or split-sample reliability testing. The NQF measure evaluation algorithm does not currently prescribe a numeric threshold for acceptable reliability.^6^ In practice, the NQF Scientific Methods Panel,^7^ which is charged with evaluating the reliability and validity of complex measures, has used 0.7 as the threshold for acceptable reliability^8,9,10,11^ and has considered 0.5 to 0.69 as borderline acceptable. These thresholds are similar to those in the Landis scale, which specifies arbitrary thresholds to quantify the measurement of observer agreement for categorical data.^12^ The Landis scale was not, however, created to evaluate measure reliability. These thresholds have not been extensively validated and are now undergoing evaluation by the NQF Scientific Methods Panel.

Adams and colleagues^9^ created a physician cost-profiling measure, and then evaluated the association between reliability and the probability that physicians’ performance would be misclassified using a 2-tier classification system. They found that the misclassification rate was lowest for the specialty with the highest measure reliability and highest for the specialty with the lowest measure reliability. Our goal was to examine the association between reliability and hospital misclassification using Centers for Medicare & Medicaid Services (CMS) hospital performance data from a broad range of performance measures commonly used in Medicare’s value-based payment programs. Because a hospital's true performance is unknown, we cannot directly measure the misclassification rate. Instead, we used the reclassification rate (eg, when hospitals switch from the upper quartile to the third quartile) as a proxy for the misclassification rate. We calculated the intraclass correlation coefficient (ICC) (test-retest reliability) and the hospital reclassification rate for each of the 28 CMS measures reported in 2017 and 2018. Although hospital reclassification across 2 different time periods may be due in part to changes in hospital performance over time instead of misclassification, the underlying cause of hospital reclassification (hospital misclassification or changes in hospital performance) will not affect the underlying association between measure reliability and hospital reclassification. We hypothesized that more reliable measures would lead to more reproducible hospital rankings across reporting periods. Our study was intended to provide empirical data based on current CMS measures that can inform efforts by the NQF, CMS, and other stakeholders to set minimal standards for measure reliability.

## Methods

### Data Sources

This study was conducted from December 13, 2020, to September 30, 2021, using data from the publicly available CMS Hospital Compare data sets 2014-2017 (hereafter, 2017 Hospital Compare) and 2015-2018 (hereafter, 2018 Hospital Compare). We selected 28 measures, including coronary artery bypass grafting mortality, chronic obstructive pulmonary disease mortality, acute myocardial infarction (AMI) mortality, pneumonia mortality, deaths among patients with complications after surgery, bloodstream infections after surgery, and heart failure readmissions (Table).^13^ We linked this data set to the CMS Impact Files data set (2017) using the hospital CMS certification number for the hospitals participating in the Inpatient Prospective Payment System. The CMS Impact Files data set included information on hospital characteristics, hospital size, resident-to-bed ratio, and average daily census.^14^

**Table.** Reclassification Rate as a Function of the Approach Used to Rank Hospitals

Measure	Hospitals, No.	Intraclass correlation coefficient (95% CI)	Reclassification rate by grouping, % (No.)			Statistical outliers, % (No.)
			Deciles	Quartiles	Terciles	
AMI mortality	2231	0.78 (0.77-0.8)	73 (1634)	45 (1014)	37 (816)	2.3 (52)
CHF mortality	3420	0.82 (0.8-0.83)	70 (2403)	43 (1485)	33 (1136)	6.3 (217)
Pneumonia mortality	3956	0.82 (0.81-0.83)	70 (2788)	44 (1742)	34 (1339)	7.8 (310)
COPD mortality	3459	0.78 (0.77-0.8)	73 (2519)	48 (1658)	36 (1260)	3.5 (121)
Stroke mortality	2473	0.82 (0.8-0.83)	71 (1757)	43 (1071)	34 (846)	4.3 (107)
AMI readmission	1769	0.81 (0.8-0.83)	71 (1261)	46 (813)	35 (619)	2.4 (43)
Hospital return days for AMI	1686	0.79 (0.77-0.81)	70 (1179)	44 (739)	35 (587)	23.4 (394)
CHF readmission	3092	0.81 (0.79-0.82)	70 (2153)	43 (1343)	35 (1082)	5.6 (172)
Hospital return days for CHF	2966	0.82 (0.81-0.83)	70 (2079)	42 (1246)	33 (992)	20.8 (618)
Pneumonia readmission	3475	0.8 (0.78-0.81)	71 (2456)	44 (1544)	34 (1197)	4.9 (170)
Hospital return days for pneumonia	3348	0.85 (0.84-0.86)	68 (2260)	39 (1318)	29 (960)	22.6 (758)
COPD readmission	2947	0.79 (0.77-0.8)	73 (2137)	47 (1397)	37 (1089)	2.1 (61)
CABG mortality	990	0.77 (0.74-0.79)	71 (699)	49 (488)	37 (371)	3.4 (34)
CABG readmission	795	0.78 (0.76-0.81)	72 (576)	48 (383)	38 (303)	2 (16)
Postoperative complications, hip and knee replacements	2673	0.75 (0.73-0.77)	70 (1880)	45 (1191)	36 (973)	4.4 (118)
Rate of readmission after hip and knee replacement	2282	0.79 (0.78-0.81)	71 (1627)	45 (1019)	36 (820)	2.5 (58)
Postoperative complications	3184	0.81 (0.79-0.82)	63 (1991)	38 (1223)	30 (946)	7.6 (242)
Failure to rescue	1676	0.73 (0.71-0.75)	76 (1268)	50 (841)	40 (663)	5.6 (94)
Postoperative respiratory failure	2685	0.77 (0.75-0.78)	65 (1740)	43 (1162)	35 (939)	6.1 (165)
Postoperative acute renal failure	2713	0.67 (0.65-0.69)	50 (1356)	30 (812)	25 (688)	1.6 (43)
Postoperative sepsis	2690	0.72 (0.7-0.74)	62 (1660)	43 (1151)	33 (899)	3.2 (87)
Dehiscence	2705	0.55 (0.53-0.58)	41 (1119)	26 (705)	21 (581)	0.6 (16)
Deep venous thrombosis	2949	0.76 (0.75-0.78)	62 (1838)	43 (1267)	34 (1006)	4.2 (123)
Pressure sores	3138	0.81 (0.79-0.82)	52 (1640)	30 (942)	25 (782)	6.6 (208)
Surgical laceration	2909	0.67 (0.65-0.69)	49 (1429)	33 (957)	27 (794)	1.1 (32)
Broken hip from a fall after surgery	3158	0.5 (0.47-0.52)	59 (1855)	37 (1173)	37 (1173)	0 (1)
Pneumothorax	3161	0.65 (0.63-0.67)	64 (2027)	33 (1042)	31 (967)	0.9 (28)
Perioperative hemorrhage	2945	0.68 (0.66-0.7)	58 (1700)	41 (1193)	32 (954)	1.8 (52)

Abbreviations: AMI, acute myocardial infarction; CABG, coronary artery bypass grafting; CHF, congestive heart failure; COPD, chronic obstructive pulmonary disease.

This study was deemed to not constitute human participant research and thus not need review by the University of Rochester Research Review Board by the vice-chair of the University of Rochester Research Subjects Review Board because the analyses were based on publicly available, hospital-level aggregated data directly downloaded from the web without a data-use agreement. The study followed the Strengthening the Reporting of Observational Studies in Epidemiology (STROBE) reporting guideline for cross-sectional studies.

### Calculation of Reliability Metrics

#### Intraclass Correlation Coefficient

We hypothesized that measures with higher ICCs would have lower reclassification rates. We based our analyses on the measures reported in the 2017 and 2018 Hospital Compare data sets. The unit of analysis was the hospital risk-standardized outcome rate. We followed the same approach for each of the 28 measures. As an illustrative example, we used the *icc* command^15^ in Stata SE/MP, version 16.1 (StataCorp LLC) to calculate the ICC for the AMI mortality measure based on all hospitals (N = 2231) (Table) with risk-standardized AMI mortality rates reported in 2017 and 2018. We used the *icc* command, which is based on a 2-way mixed-effects model (*icc, mixed*) in which every hospital is rated using the same 2 raters: the risk-standardized rates reported in 2017 and 2018. We specified the ICC so that the consistency of agreement of the measures was estimated (this is the default for the *icc, mixed* command), meaning to what extent do the 2 raters agree on the rankings of the hospitals. The 2-way linear mixed-effect model used to estimate the ICC is specified as follows^15^:

y_ij_ = μ + h_i_ + m_j_ + ε_ij_

where *y_ij_* is the AMI risk-standardized mortality rate for hospital *i* by rater *j* (1, based on 2017 data; and 2, based on 2018 data), μ is the mean hospital risk-standardized mortality rate, *h_i_* is the hospital random effect, and *m_j_* is a fixed effect and refers to whether the risk-standardized mortality rate is based on the 2017 or the 2018 data. In this case, the ICC was defined as follows:

ICC = σ^2^*_h_*/(σ^2^*_h_* + σ^2^_ε_)

where σ^2^*_h_* is the variance of the hospital random effects term and σ^2^_ε_ is the variance of the error term. We calculated the ICC for each of the 27 other measures using this same approach.

#### Reclassification Rate and κ Statistic

Hospitals were ranked by their AMI risk-standardized mortality rate using the 2017 data set into 10 equal-sized deciles. The hospitals were then separately ranked into deciles based on their AMI risk-standardized mortality rate in the 2018 data set. We calculated the reclassification rate as the proportion of hospitals that switched from one decile in the 2017 ranking to a different decile in 2018. We examined the interrater agreement for the decile rankings based on the 2017 and 2018 data sets using the κ statistic. The κ statistic quantifies the level of agreement in the hospital rankings based on the 2017 and 2018 data after correcting for the level of agreement that would occur due to chance. We used this same approach after categorizing the hospitals into quartiles and terciles, and using the performance categories based on statistical outlier status (high, low, and average performance). We repeated this approach for each of the 28 measures separately. eTable 1 in the Supplement lists the classification approaches used by the CMS in public reporting and value-based purchasing.

### Statistical Analysis

We used bivariate linear regression analyses to examine the association between the reclassification rate and the ICC, and the reclassification rate and the κ statistic for the hospitals categorized into deciles. We repeated these analyses with hospitals categorized into quartiles, terciles, and statistical outliers (high, low, and average performance).

We performed secondary analyses in which we excluded hospitals with either less than 250 cases in the 2017 data set or less than 500 cases in the 2017 data set. We performed these secondary analyses because the point estimates for the performance measures may be less stable when these estimates are based on small numbers of cases, despite the use of shrinkage estimators in the CMS measures.

Next, we performed a post hoc analysis in which we examined the association between the reclassification rate and the number of groups used to rank hospitals controlling for the ICC. We conducted these analyses after observing that the reclassification rate decreased as the number of groups used to rank hospitals became smaller.

Data management and statistical analyses were performed using Stata SE/MP, version 16.1. All statistical tests were 2-tailed, and *P* values <.05 were considered significant. We used robust variance estimators to account for possible heteroskedasticity of the error terms.

## Results

### Study Sample

**Figure 1.**
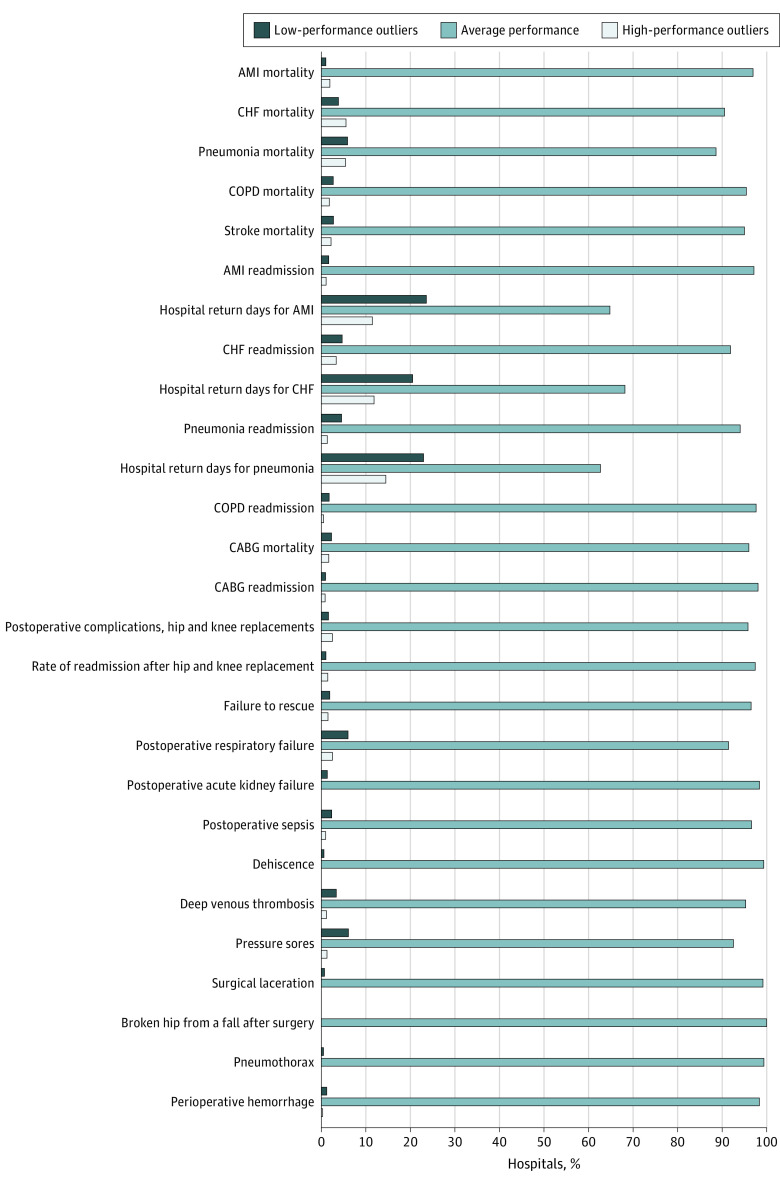
Distribution of Low-Performance, High-Performance, and Average-Performance Hospitals for Centers for Medicare & Medicaid Services (CMS) Performance Measures Based on Analysis of CMS Hospital Compare Data AMI indicates acute myocardial infarction; CABG, coronary artery bypass grafting; CHF, congestive heart failure; COPD, chronic obstructive pulmonary disease.

**Figure 2.**
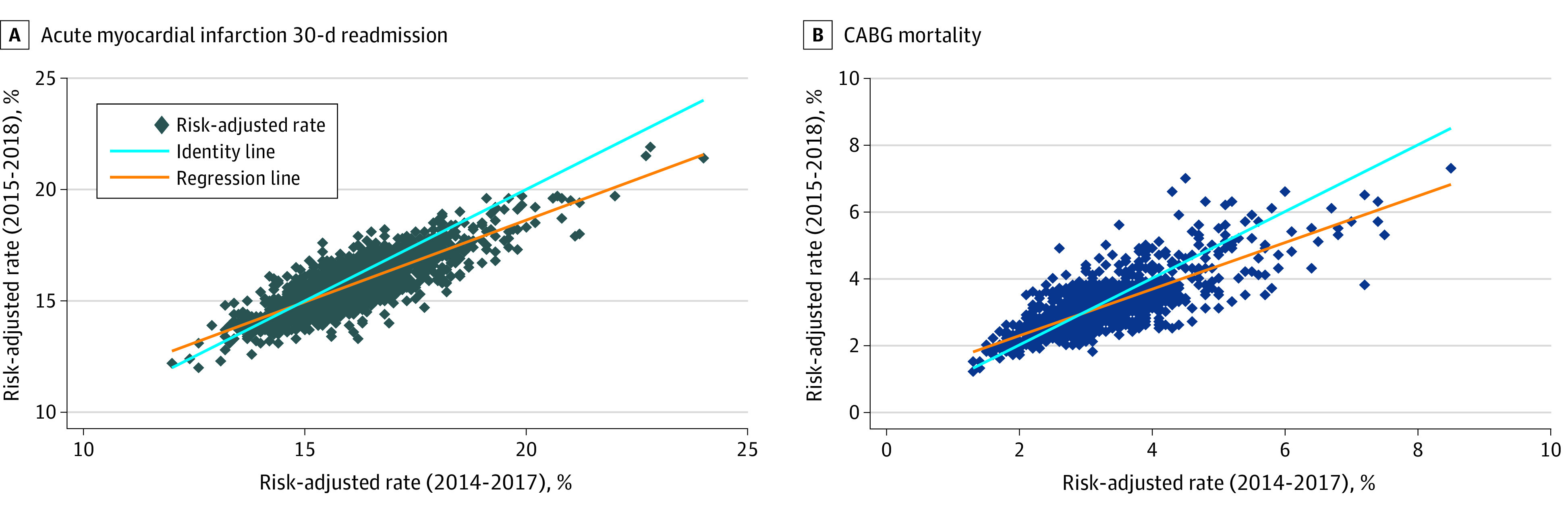
Comparison of Risk-Adjusted Hospital Acute Myocardial Infarction 30-Day Readmission and Coronary Artery Bypass Grafting (CABG) Risk-Adjusted 30-Day Mortality Rates, Using the CMS Hospital Compare Data Sets: 2014-2017 vs 2015-2018 Diamonds represent individual hospitals.

The analytic cohort consisted of 28 CMS Hospital Compare measures based on 4452 hospitals, with a median of 2927 (IQR, 2378-3160) hospitals contributing data for each measure. The hospitals participating in the Inpatient Prospective Payment System (n = 3195) had median bed size of 141 (IQR, 69-261), average daily census of 70 (IQR, 24-155) patients, and 38.2% (IQR, 18.7%-36.6%) disproportionate share hospital percentage. Our analysis was based on 28 different performance measures, including mortality measures (eg, AMI, congestive heart failure, pneumonia, and coronary artery bypass grafting), readmission measures (eg, AMI, congestive heart failure, pneumonia, and coronary artery bypass grafting), and surgical complications (eg, postoperative acute kidney failure, postoperative respiratory failure, postoperative sepsis, and failure to rescue). The median percentage of hospitals identified as average performance per measure was 96.0% (IQR, 92.0%-98.2%) (Figure 1). The median ICC for the measures was 0.78 (IQR, 0.72-0.81). Findings for each of the measures are reported in the Table. The variation in hospital performance scores across reporting periods (2017 and 2018) are shown in Figure 2 and eFigure 2 in the Supplement. The median reclassification rate was 70.0% (IQR, 62.0%-71.2%) when hospitals were ranked by deciles, 43.4% (IQR, 38.9%-45.1%) when ranked by quartiles, 34.3% (IQR, 31.5%-36.4%) when ranked by terciles, and 3.8% (IQR, 2.0%-6.2%) when ranked by statistical outlier status. Seventy-nine percent of the CMS Hospital Compare measures exceeded the commonly used reliability threshold of 0.7. The reclassification rate for these measures was 69% when hospitals were ranked by deciles, 44% when ranked by quartiles, and 34% when ranked by terciles.

### Association of Reclassification Rate and the Approach Used to Measure Reliability

#### Intraclass Correlation Coefficient

**Figure 3.**
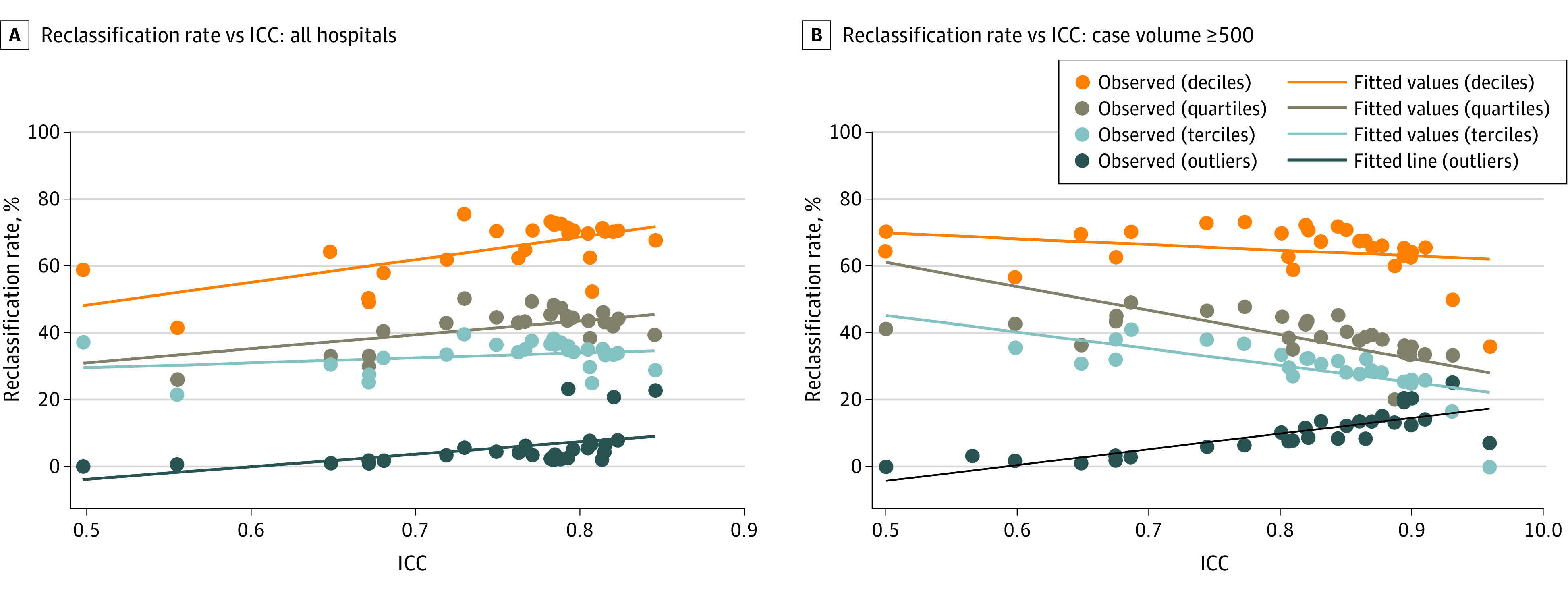
Results of Bivariate Linear Regression Examining the Association Between the Reclassification Rate and the Intraclass Correlation Coefficient (ICC) for the Hospitals Categorized Into Deciles, Quartiles, Terciles, and Based on Outlier Status A, All hospitals. B, Hospitals with case volumes with 500 or more cases. Each of the points represents a separate hospital performance measure (eg, risk-adjusted 30-day mortality rate after acute myocardial infarction).

In the baseline analysis, we unexpectedly found that increases in the ICC were associated with increases in the reclassification rate (Figure 3A; eTable 2 in the Supplement). Each 0.1-point increase in the ICC was associated with a 6.80 (95% CI, 2.28-11.30; *P* = .005) percentage-point increase in the reclassification rate when hospitals were ranked into performance deciles, 4.15 (95% CI, 1.16-7.14; *P* = .008) when ranked into performance quartiles, 1.47 (95% CI, 1.84-4.77; *P* = .37) when ranked into performance terciles, and 3.70 (95% CI, 1.30-6.09; *P* = .004) when ranked by outlier status. After excluding hospitals with case volumes less than 500 cases, each 0.1-point increase in the ICC was associated with a 1.72 (95% CI, −5.24 to 1.79; *P* = .32) percentage point decrease when hospitals were ranked into performance deciles, 7.19 percentage point decrease (95% CI, −11.30 to −3.07; *P* = .001) when ranked into performance quartiles, 4.98 (95% CI, −7.80 to −2.17; *P* = .001) percentage point decrease when ranked into performance terciles, and 4.70 (95% CI, 3.02-6.39; *P* < .001) percentage point increase when hospitals were ranked by outlier status (Figure 3B; eTable 2 in the Supplement). The median number of hospitals per measure was 2927 (IQR, 2378-3160). Excluding hospitals with case volumes less than 500 led to the exclusion of a median of 1687 (IQR, 1086-2453) hospitals per measure (eFigure 1 in the Supplement).

#### κ Statistic

**Figure 4.**
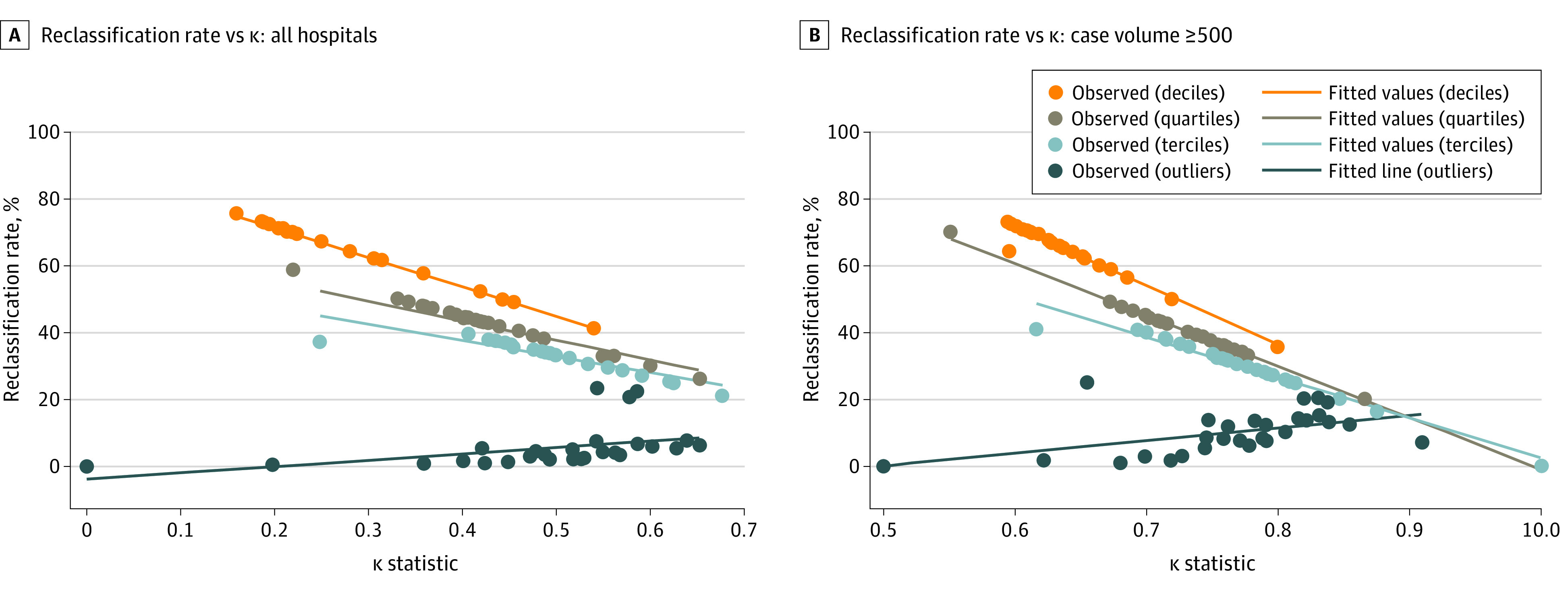
Results of Bivariate Linear Regression Examining the Association Between the Reclassification Rate and the κ Statistic for the Hospitals Categorized Into Deciles, Quartiles, Terciles, and Based on Outlier Status Each of the points represents a separate hospital performance measure (eg, risk-adjusted 30-day mortality rate after acute myocardial infarction).

We saw significant decreases in the reclassification rate as the κ statistic increased (Figure 4; eTable 2 in the Supplement). The reclassification rate decreased by 8.78 (−9.25 to −8.32; *P* < .001) percentage points for every 0.1-point increase in the κ statistic when hospitals were ranked in deciles, 5.86 (95% CI, −8.87 to −2.85; *P* < .001) when ranked in quartiles, and 4.84 (95% CI, −7.39 to 2.29; *P* < .001) when ranked in terciles.

#### Changes in Reclassification Rate as a Function of Ranking Method

We found that the method used to rank hospitals (deciles, quartiles, or terciles) had a greater association with the reclassification rate than changes in the ICC (Figure 3). In a post hoc analysis, we found that ranking hospitals by deciles, quartiles, and terciles led to a higher percentage-point reclassification rate compared with ranking hospitals by outlier status, after controlling for the ICC (deciles: 59.9; 95% CI, 56.6-63.2; *P* < .001; quartiles: 35.9; 95% CI, 33.1-38.8; *P* < .001; and terciles: 27.6; 95% CI, 24.9-30.3; *P* < .001) (eTable 3 in the Supplement). We also found that the reclassification rate increased when hospitals were ranked by outlier status as the ICC increased in both the baseline and sensitivity analyses (Figure 3; eTable 2 in the Supplement). We found similar results when this analysis was based on the κ statistic (Figure 4 and eTable 2 in the Supplement).

## Discussion

We noted that more reliable quality measures were not less likely to reclassify hospitals compared with less reliable measures when reliability was assessed using test-retest reliability testing. Instead, we found that the most important factor associated with the reclassification rate was the approach used to rank hospital performance. The reclassification rate was highest when hospitals were ranked by deciles and lowest when hospitals were ranked based on their outlier status. The larger the number of categories used to rank hospitals the more likely hospitals will switch categories when they are ranked a second time. In other words, the reproducibility of hospital rankings is partly a function of the hospital ranking system. Although not commonly appreciated, the association between the number of categories used to rank hospitals and the reclassification rate is as expected, because the greater the number of performance categories, the greater the number of opportunities for hospitals to switch categories. To our knowledge, the magnitude of this association has not previously been described. It is notable that the CMS classifies hospitals in the Hospital Value-based Purchasing,^16^ the Comprehensive Care for Joint Replacement Program,^17^ and physicians in the Merit-based Incentive Payment System^18^ using deciles (eTable 1 in the Supplement).

We also noted that, although 79% of the CMS Hospital Compare measures exceeded the commonly used reliability threshold of 0.7, the reclassification rate for measures meeting this threshold was 69% when hospitals were ranked by deciles, 44% when ranked by quartiles, and 34% when ranked by terciles. These values may appear to be unrealistically high at first but are, in fact, reasonable after recognizing the substantial variability in the point estimates for the hospital risk-standardized rates across the 2 reporting periods as shown in Figure 2 and eFigure 2 in the Supplement for each of the 28 measures, even for measures with a high ICC. In sensitivity analyses, we found, as expected, that the reclassification rate decreased as reliability increased after we excluded hospitals with fewer than 500 cases and hospitals were ranked into quartiles and deciles. However, because the CMS does not exclude hospitals with fewer than 500 cases (which constitute more than half of hospitals),^19^ the finding that higher reliability leads to a lower-reclassification rate in high-volume hospitals has limited utility.

We noted that hospitals shifted rankings only 3.8% of the time when classified into 3 categories (high, average, and low performance) based on whether they were statistical outliers, and they shifted 34% of the time when they were ranked into 3 equal-sized terciles. This outcome was not unexpected because most hospitals are considered average when statistical criteria are used to classify hospitals as high, average, and low performance. When nearly all hospitals are classified as average performance, it is not possible for a large proportion of hospitals to shift categories.

Because the ICC could not be used to identify measures with low reclassification rates, we decided to examine whether the κ statistic could be used instead to quantify reliability. We used the κ statistic to examine agreement in the hospital rankings in 2017 and 2018 because it corrects for the amount of agreement that would be expected by chance. We found that measures with higher κ statistic values had lower reclassification rates. The reclassification rate decreased by nearly 9 percentage points for each 0.1-percentage-point increase in the κ statistics when hospitals were classified into performance deciles. However, measures with a high κ statistic value have low reclassification rates because the κ statistic represents the interrater agreement (the extent to which hospital rankings in 2017 are similar to hospital rankings in 2018) adjusted for the expected agreement.^20^

Our findings suggest that better performance on test-retest reliability testing does not mean that hospital rankings are more reproducible. By definition, a performance measure is considered reliable if repeated measures of the performance of the same hospitals yield similar results (ie, hospital ranks). Reliability is one of the essential criteria used by the NQF to assess scientific acceptability of performance measures submitted for endorsement. Poor reliability is akin to using a yardstick to measure the outside perimeter of a room and getting very different measures each time. Our findings suggest that better performance on test-retest reliability testing cannot be used to identify performance measures that yield more reproducible results.

In addition, the extent to which hospitals change rankings across 2 reporting periods is striking and raises questions regarding the validity of basing public reporting and value-based purchasing on hospital rankings. Seventy percent of hospitals changed ranks when hospitals were ranked by deciles, and 34% when ranked by terciles. It is unlikely that true changes in hospital performance accounted for such a large shift in rankings. The finding that hospital performance can vary substantially for the same outcome when different risk-adjustment models are used was first shown in the seminal work by Iezzoni^21^ and has been replicated by others.^22,23^ But in the case of the CMS Hospital Compare measure, we are comparing hospital rankings based on the same risk-adjustment model and showing very substantial shifts in ranking across overlapping periods. However, hospital-adjusted outcomes have been shown to estimate the probability of future hospital performance,^10,24,25,26^ and the technical skills of surgeons are associated with better surgical risk-adjusted outcomes.^27^ Taken together, these findings suggest that risk-adjusted rates measure quality, but that the rankings of individual hospitals based on these rates should be interpreted with caution.

### Limitations

This study has limitations. First, we had access to hospital-level performance score data and not to the patient-level data used to create these measures. Because of this lack of data, we only examined test-retest reliability and did not examine the association between the signal-to-noise ratio and the reclassification rate. Second, our analysis was limited to test-retest reliability testing instead of the more commonly used split-sample reliability testing. With split-sample reliability testing,^6,11^ the hospital cases for a single time period are split into halves. Each hospital's performance is separately measured using each of the 2 samples. However, our analysis of the association between the ICC and reclassification did not depend on whether the measure scores were calculated in the same time period (as is the case for split-sample reliability testing) or in separate time periods (as is the case for test-retest reliability testing). The results of the analysis are based only on the hospital risk-standardized outcomes in each of the 2 hospital samples, and not on the method used to generate the 2 samples. Third, because hospital quality may have changed over time, the hospital reclassification rate may not be a good proxy for misclassification. However, our analysis examined the association between the ICC and the reclassification rate, and our findings would apply equally well to split-sample reliability testing where the hospital reclassification rate would be a good proxy for misclassification. Fourth, our results cannot be generalized to reliability testing based on the signal-to-noise ratio, because this approach is distinctly different from test-retest reliability or split-sample reliability testing.

## Conclusions

Although measures are generally considered reliable if the reliability is 0.7 or greater, there is nearly no empirical justification for this threshold. Our analysis of CMS hospital performance measures found little evidence that measures assessed as more reliable using test-retest reliability testing were less likely to reclassify hospitals in a subsequent period. However, we found that the κ statistic, which is also a measure of interrater agreement, was correlated with the reclassification rate. Our findings suggest that measure reliability should not be assessed with test-retest or split-sample reliability testing. Additional work is necessary to investigate the validity of the signal-to-noise ratio for assessing measure reliability.

## Notes

Supplement. **eTable 1.** Ranking of Hospitals and Physicians in CMS Public Reporting and Value-based Purchasing **eTable 2.** Reclassification as a Function of the Intraclass Correlation Coefficient or the κ Statistic **eTable 3.** Association Between the Reclassification Rate and the Number of Performance Categories, Controlling for the ICC **eFigure 1.** Distribution of Hospitals With Fewer Than 250 Cases, 250-499 Cases, and 500 or More Cases for Each of the Performance Measures During the 2014-2107 Reporting Period for the CMS Hospital Compare Measures **eFigure 2.** Comparison of Hospital Risk-adjusted Rates in the CMS Hospital Compare Datasets: 2014-2017 versus 2015-2018
