# Supplementary material for: Reproducibility of Hospital Rankings Based on Centers for Medicare & Medicaid Services Hospital Compare Measures as a Function of Measure Reliability
Source: JAMA Netw Open. 2021 Dec 7;4(12):e2137647. doi: 10.1001/jamanetworkopen.2021.37647 (PMC8652605; doi:10.1001/jamanetworkopen.2021.37647)
Supplement: Supplement. — eTable 1. Ranking of Hospitals and Physicians in CMS Public Reporting and Value-based Purchasing eTable 2. Reclassification as a Function of the Intraclass Correlation Coefficient or the κ Statistic eTable 3. Association Between the Reclassification Rate and the Number of Performance Categories, Controlling for the ICC eFigure 1. Distribution of Hospitals With Fewer Than 250 Cases, 250-499 Cases, and 500 or More Cases for Each of the Performance Measures During the 2014-2107 Reporting Period for the CMS Hospital Compare Measures eFigure 2. Comparison of Hospital Risk-adjusted Rates in the CMS Hospital Compare Datasets: 2014-2017 versus 2015-2018 [file jamanetwopen-e2137647-s001.pdf]

Supplementary Online Content

Glance LG, Nerenz DR, Joynt Maddox KE, Hall BL, Dick AW. Reproducibility of hospital rankings based on Centers for Medicare & Medicaid services hospital compare measures as a function of measure reliability. *JAMA Netw Open*. 2021;4(12):e2137647. doi:10.1001/jamanetworkopen.2021.37647

eTable 1. Ranking of Hospitals and Physicians in CMS Public Reporting and Value-based Purchasing

eTable 2. Reclassification as a Function of the Intraclass Correlation Coefficient or the κ Statistic

eTable 3. Association Between the Reclassification Rate and the Number of Performance Categories, Controlling for the ICC

eFigure 1. Distribution of Hospitals With Fewer Than 250 Cases, 250-499 Cases, and 500 or More Cases for each of the performance measures during the 2014-2107 reporting period for the CMS Hospital Compare measures

eFigure 2. Comparison of Hospital Risk-adjusted Rates in the CMS Hospital Compare Datasets: 2014-2017 versus 2015-2018

This supplementary material has been provided by the authors to give readers additional information about their work.

© 2021 Glance LG et al. *JAMA Network Open.*

eTable 1. Ranking of Hospitals and Physicians in CMS Public Reporting and Value-based Purchasing

	Number of Categories	Method used to Classify Hospitals or Physicians	Source
Hospital Compare Star Rating	5	k-means clustering bins hospitals into 5 groups based on average scores	https://jamanetwork.com/journals/jama/fullarticle/2780295
Hospital Compare Complications and Deaths	3	outlier status based on 95% confidence interval	https://data.cms.gov/provider-data/topics/hospitals/complications-deaths
Hospital Readmission Reduction Program	continuous	excess readmission ratio (analogous to OE ratio)(continuous)	https://qualitynet.cms.gov/inpatient/hrrp/methodology
Hospital-Acquired Condition Reduction Program	quartiles	quartiles	https://www.cms.gov/Medicare/Medicare-Fee-for-Service-Payment/AcuteInpatientPPS/HAC-Reduction-Program
Hospital Value-Based Purchasing Program	50th percentile; deciles	50th percentile; deciles	https://www.bmj.com/content/bmj/suppl/2016/05/09/bmj.i2214.DC1/figj030884.ww1.pdf
Comprehensive Care for Joint Replacement (CJR) Program	deciles	deciles	https://innovation.cms.gov/files/x/cjr-qualsup.pdf
Medicare Shared Savings Plan (accountable care organizations)	continuous	MIPS quality points (continuous)	https://www.cms.gov/files/document/medicare-shared-savings-program-shared-savings-and-losses-and-assignment-methodology-specifications.pdf-0
Merit-based Incentive Payment System (MIPS)	deciles	deciles	https://www.hhs.gov/guidance/sites/default/files/hhs-guidance-documents/2019%20MIPS%20Quality%20Performance%20Category%20Factsheet_3.pdf

© 2021 Glance LG et al. *JAMA Network Open.*

eTable 2. Reclassification as a Function of the Intraclass Correlation Coefficient or the κ Statistic

Appendix Table 2. Reclassification as a function of the Intraclass Corelation Coefficient or the Kappa statistic

	Baseline			>= 250 cases			>= 500 cases		
	coefficient	95% CI	P	coefficient	95% CI	P	coefficient	95% CI	P
Intraclass Correlation Coefficient									
decile	6.80	(2.28,11.31)	0.005	3.84	(1.54,6.14)	0.002	-1.72	(-5.24,1.79)	0.323
quartile	4.15	(1.2,7.1)	0.008	0.81	(-0.67,2.3)	0.271	-7.19	(-11.31,-3.07)	0.001
tercile	1.47	(-1.84,4.77)	0.370	-1.21	(-3.16,0.73)	0.212	-4.98	(-7.8,-2.17)	0.001
outliers	3.70	(1.3,6.09)	0.004	4.80	(2.91,6.69)	<0.001	4.70	(3.02,6.39)	<0.001
Kappa									
decile	-8.78	(-9.25,-8.32)	<0.001	-8.58	(-9.37,-7.79)	<0.001	-8.76	(-9.57,-7.96)	<0.001
quartile	-5.86	(-8.87,-2.85)	<0.001	-4.62	(-8.79,-0.45)	<0.001	-7.69	(-8.03,-7.35)	<0.001
tercile	-4.84	(-7.39,-2.29)	<0.001	-4.66	(-6.92,-2.4)	<0.001	-6.01	(-7.2,-4.82)	<0.001
outliers	1.92	(0.67,3.17)	0.004	2.90	(1.3,4.5)	<0.001	1.88	(0.27,3.49)	0.024

Results of bivariate linear regression examining the association between the re-classification rate and the (1) intraclass correlation coefficient, and (2)) kappa statistic for the hospitals categorized into deciles, quartiles, terciles, and based on outlier status. Each row in the Table shows the slope coefficient for a separate bivariate regression model (e.g. the 1st row shows the reclassification rate as a function of the ICC when hospitals are categorized into deciles). The values for the intercepts are not shown. The coefficients represent the marginal change in the reclassification rate when the ICC or kappa statistic increase by 0.1.

abbreviations: CI – confidence interval

© 2021 Glance LG, et al. *JAMA Network Open.*

eTable 3. Association Between the Reclassification Rate and the Number of Performance Categories, Controlling for the ICC

Appendix Table 3. Association between the reclassification rate and the number of peformance categories, controlling for the ICC

	Coefficient (95% CI)		P value	adj R-sqared
Category				
Statisitical outliers	reference			
Terciles	27.6	(24.9,30.3)	<0.001	
Quartiles	35.9	(33.1,38.8)	<0.001	
Deciles	59.9	(56.6,63.2)	<0.001	
ICC	40.3	(22.6,58)	<0.001	
constant	-24.7	(-37.8,-11.6)	<0.001	0.94

Results of multivariate linear regression examining the association between the re-classification rate and the method used to rank hospitals, controlling for the ICC.

abbreviations: CI – confidence interval; ICC – intraclass correlation coefficient

© 2021 Glance LG, et al. *JAMA Network Open.*

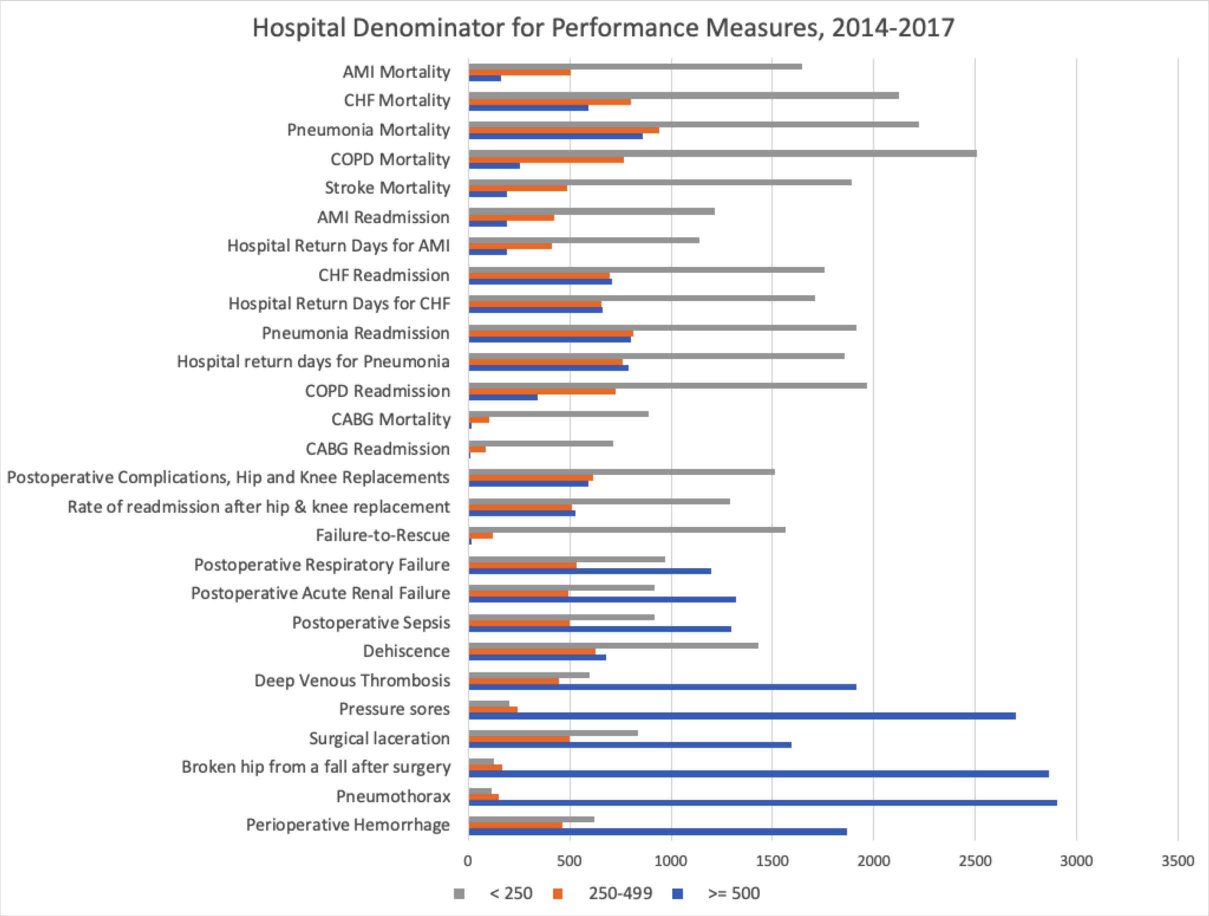

eFigure 1. Distribution of hospitals with fewer than 250 cases, 250-499 cases, and 500 or more cases for each of the performance measures during the 2014-2107 reporting period for the CMS Hospital Compare measures.

© 2021 Glance LG, et al. *JAMA Network Open.*

eFigure 2. Comparison of hospital risk-adjusted rates in the CMS Hospital Compare datasets: 2014-2017 versus 2015-2018.

eFigure 2.1

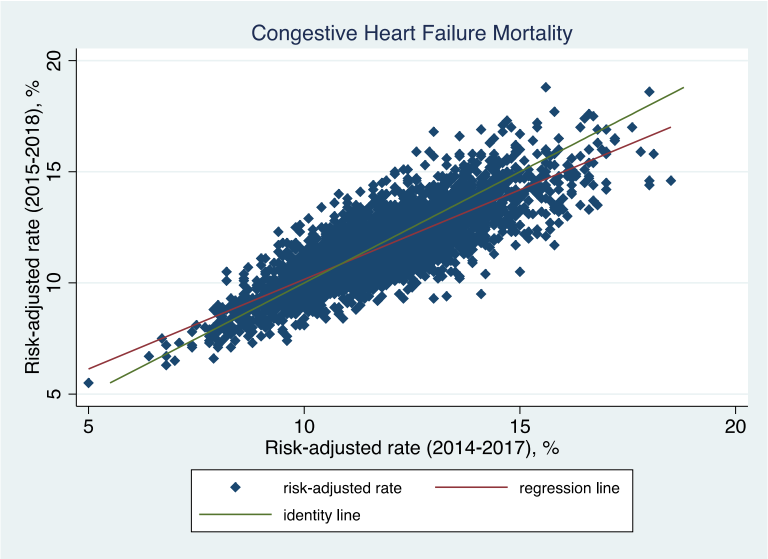

eFigure 2.2

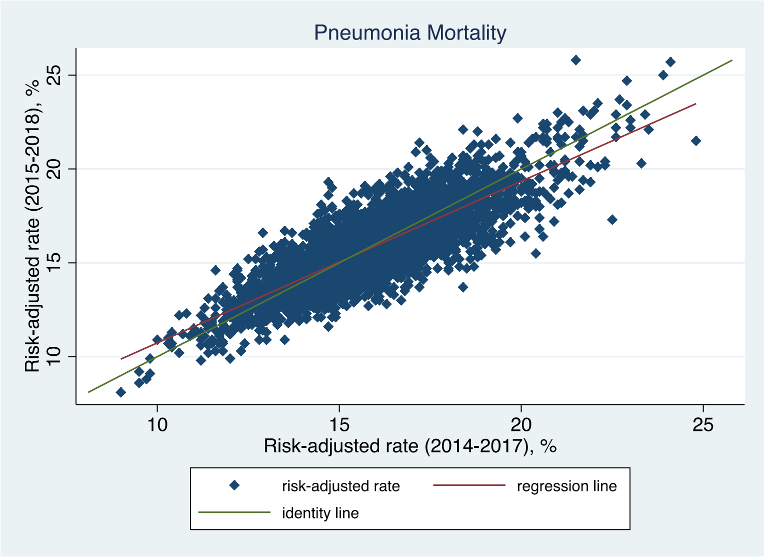

© 2021 Glance LG, et al. *JAMA Network Open.*

eFigure 2.3

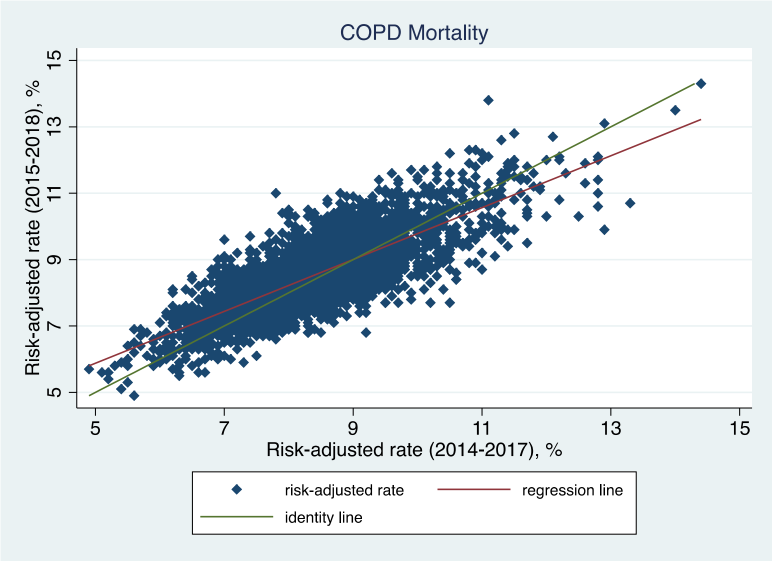

eFigure 2.4

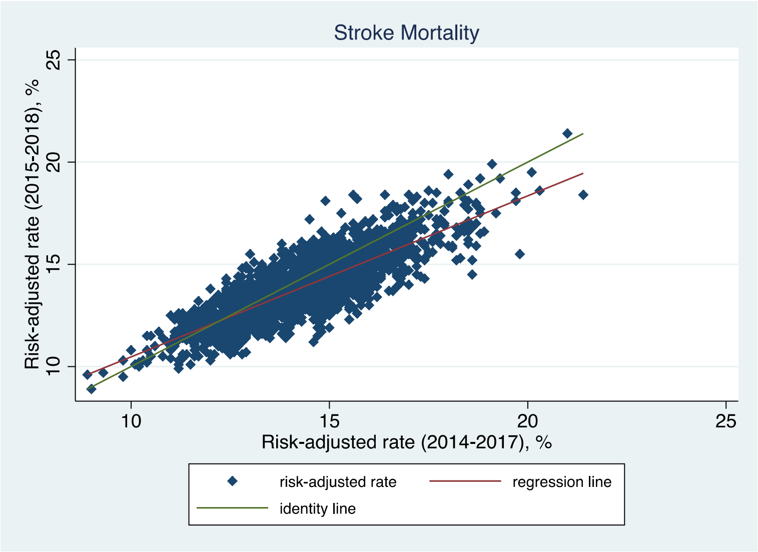

© 2021 Glance LG, et al. *JAMA Network Open.*

eFigure 2.5

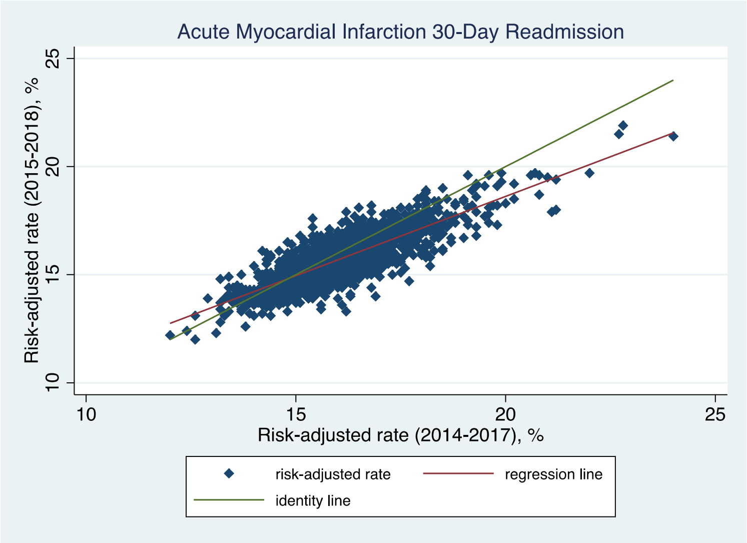

eFigure 2.6

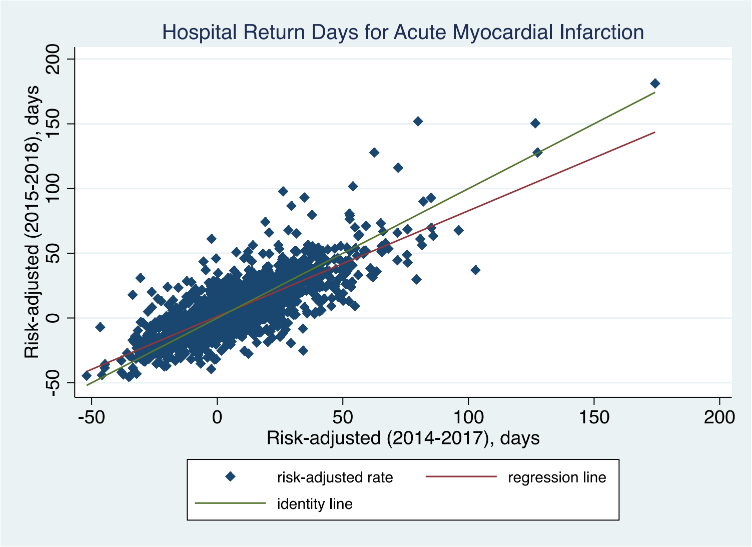

© 2021 Glance LG, et al. *JAMA Network Open.*

eFigure 2.7

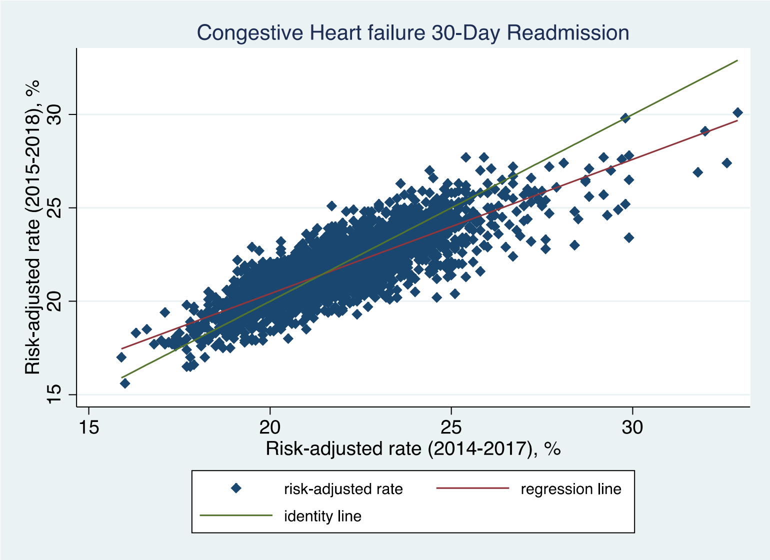

eFigure 2.8

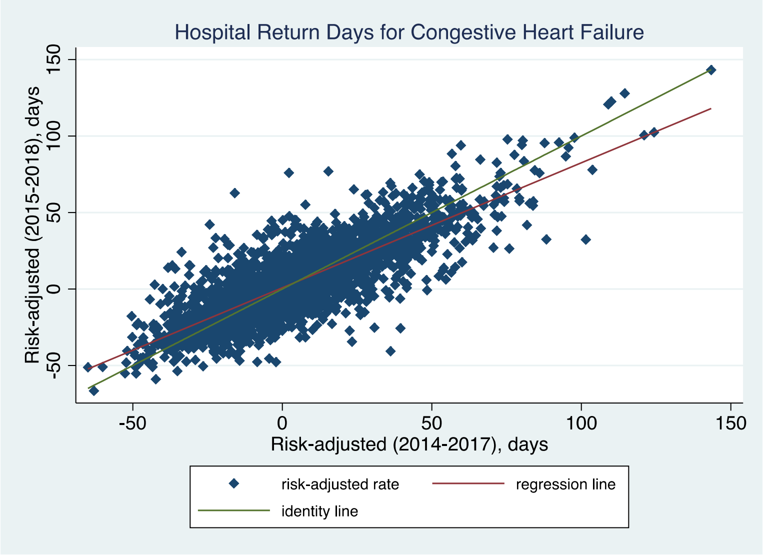

© 2021 Glance LG, et al. *JAMA Network Open.*

eFigure 2.9

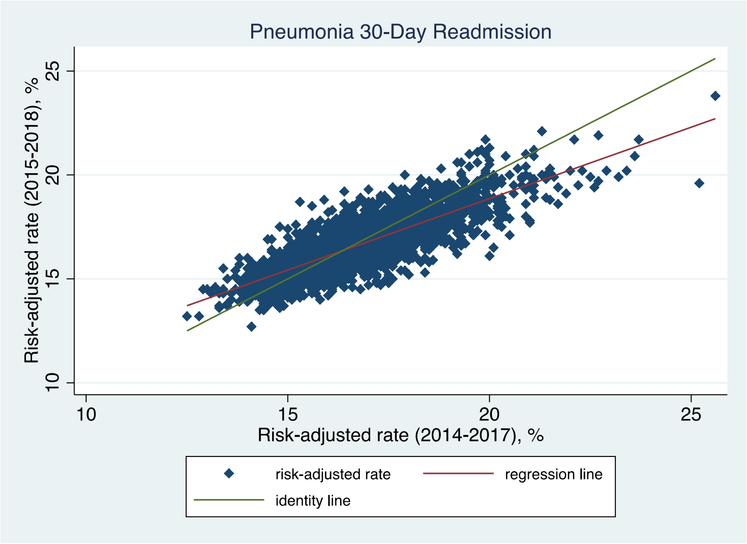

eFigure 2.10

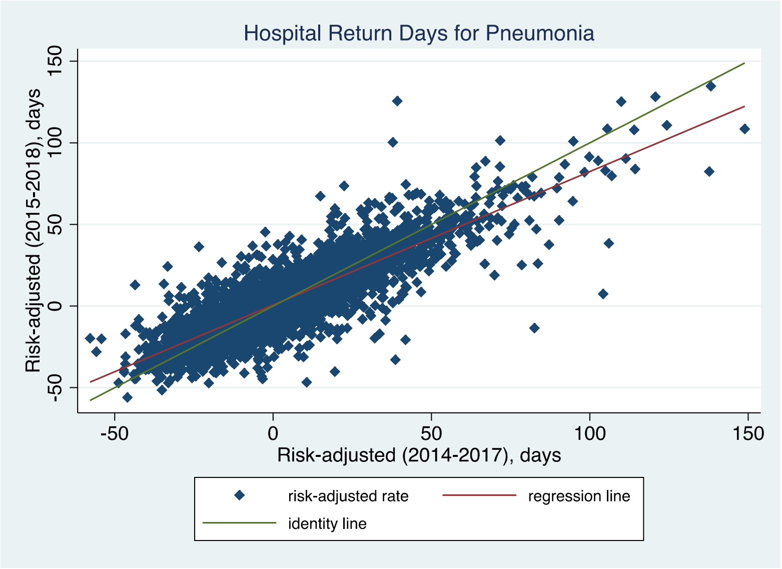

© 2021 Glance LG, et al. *JAMA Network Open.*

eFigure 2.11

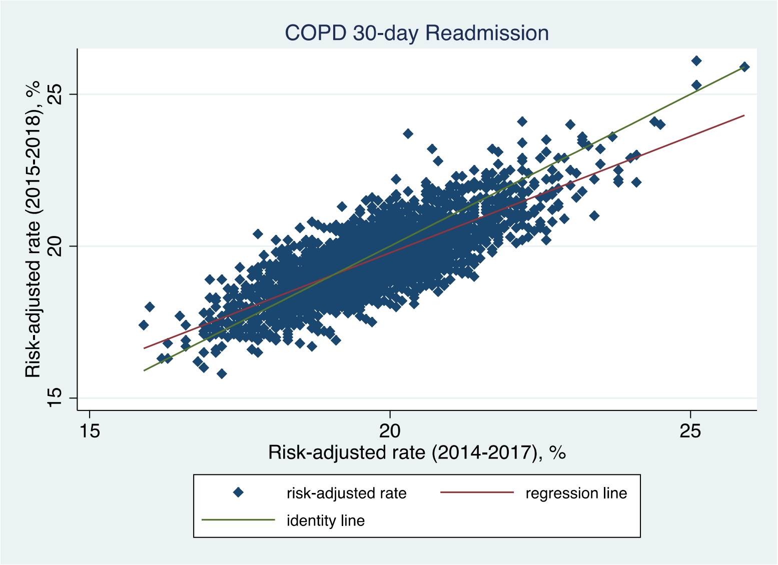

eFigure 2.12

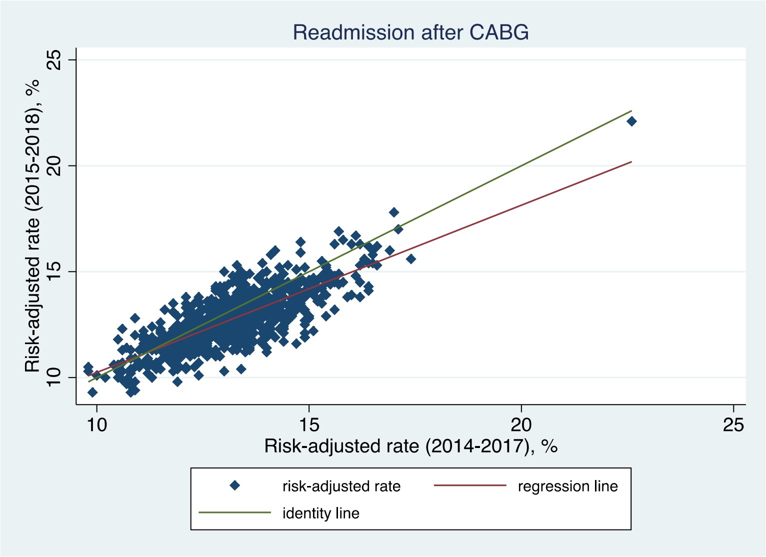

© 2021 Glance LG, et al. *JAMA Network Open.*

eFigure 2.13

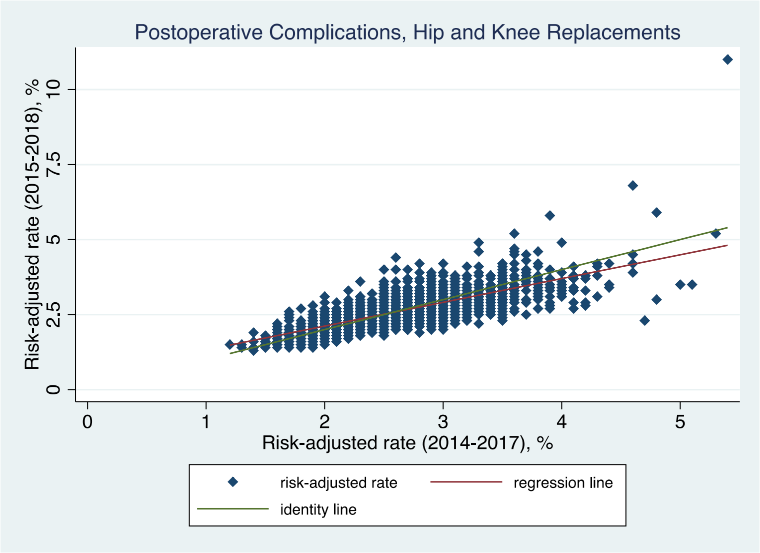

eFigure 2.14

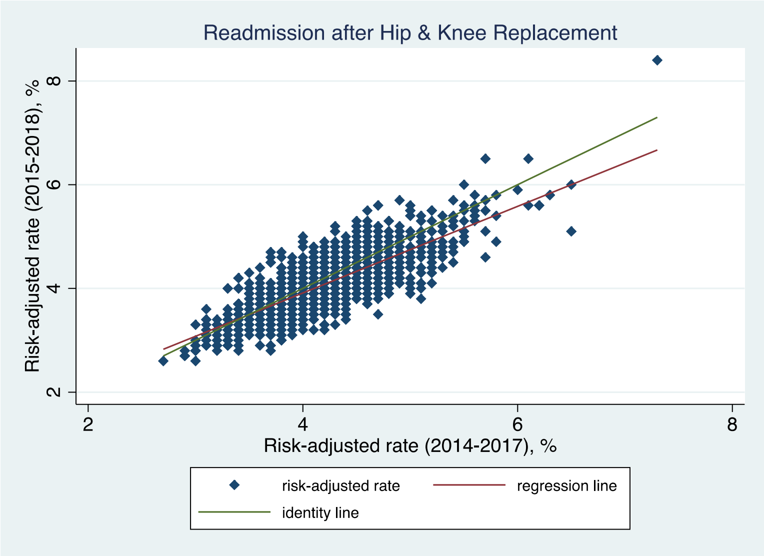

© 2021 Glance LG, et al. *JAMA Network Open.*

eFigure 2.15

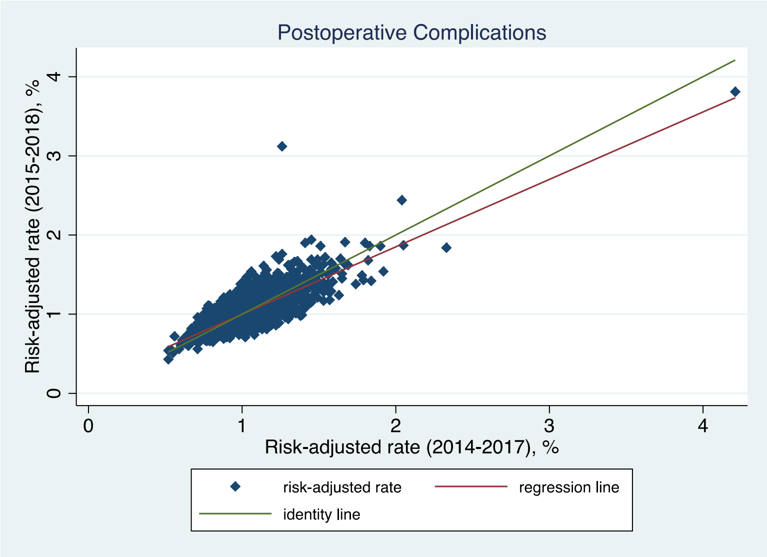

eFigure 2.16

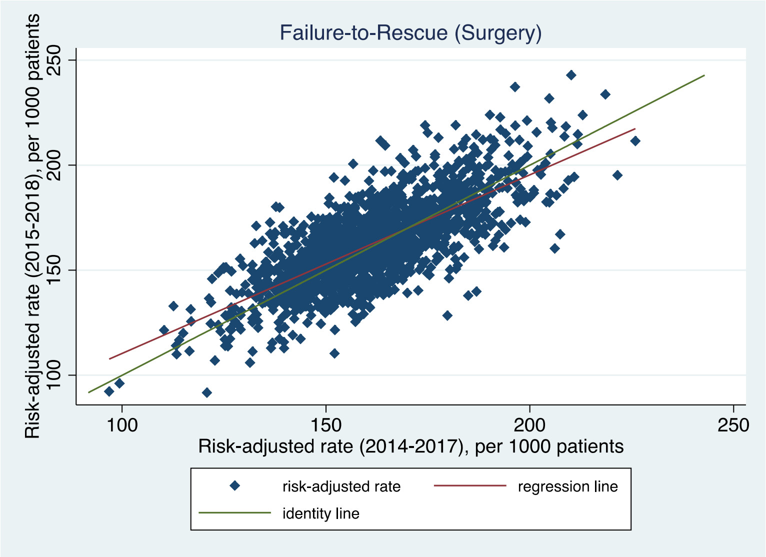

© 2021 Glance LG, et al. *JAMA Network Open.*

eFigure 2.17

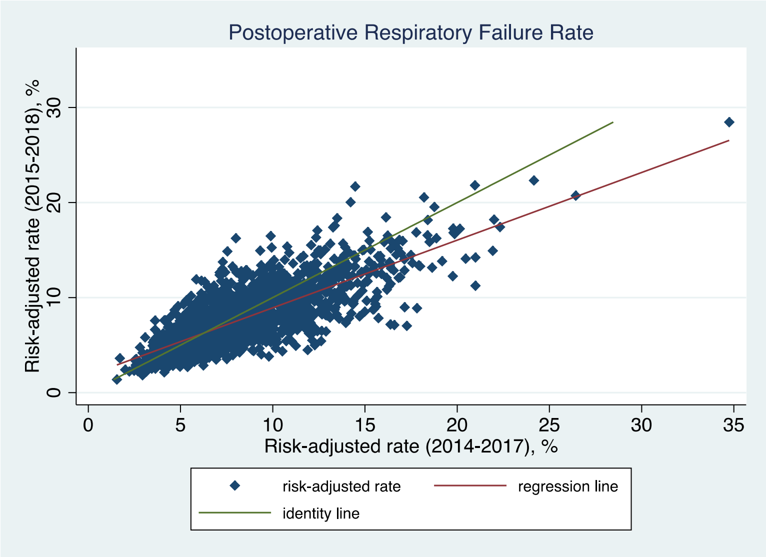

eFigure 2.18

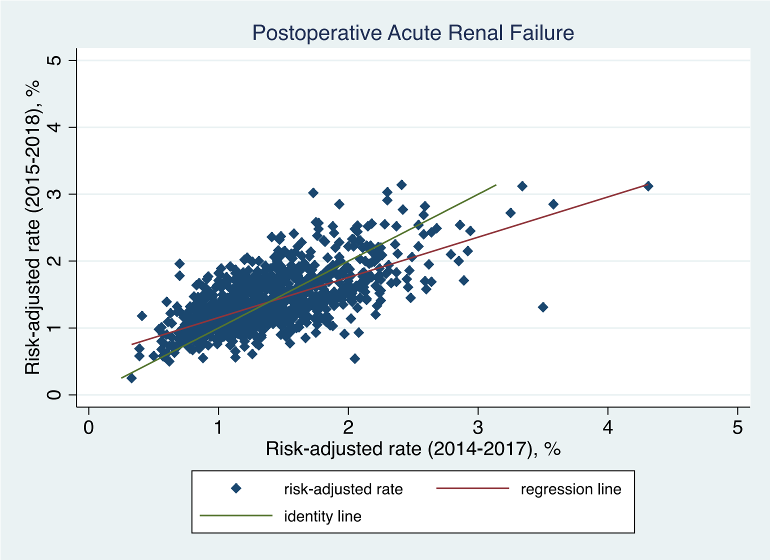

© 2021 Glance LG, et al. *JAMA Network Open.*

eFigure 2.19

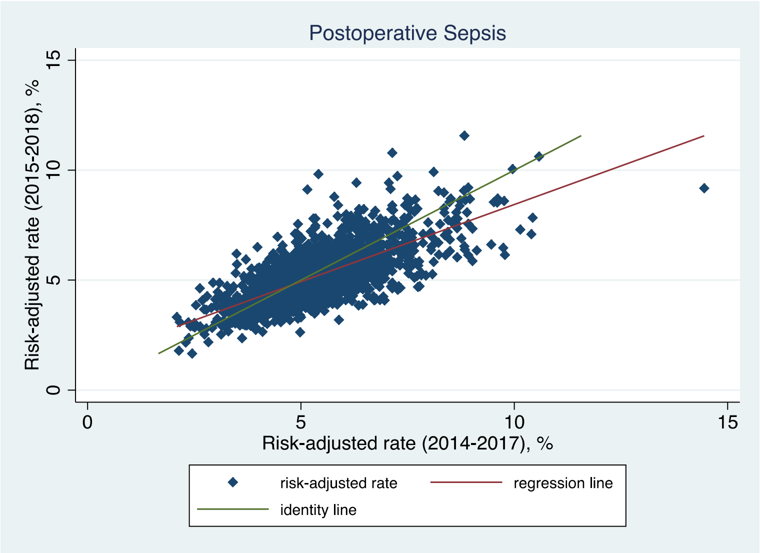

eFigure 2.20

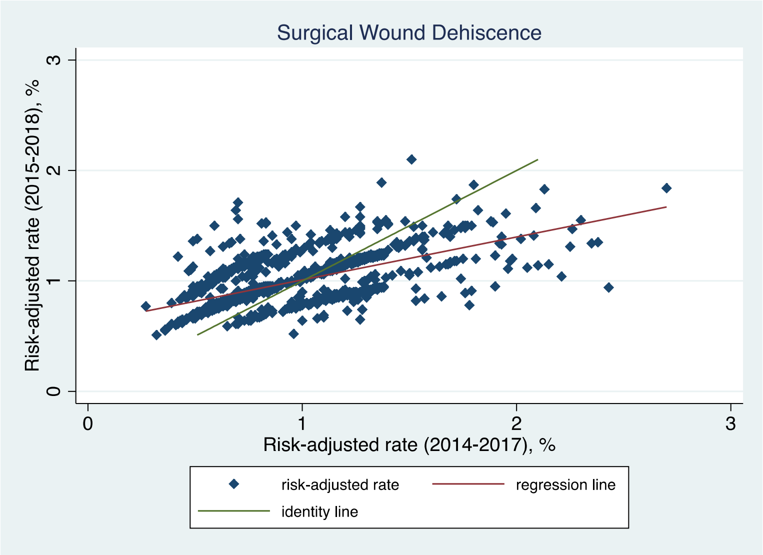

© 2021 Glance LG, et al. *JAMA Network Open.*

eFigure 2.21

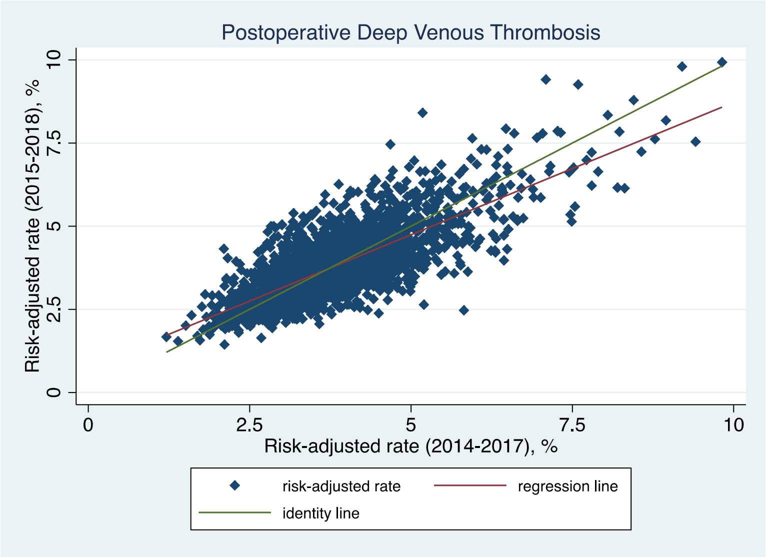

eFigure 2.22

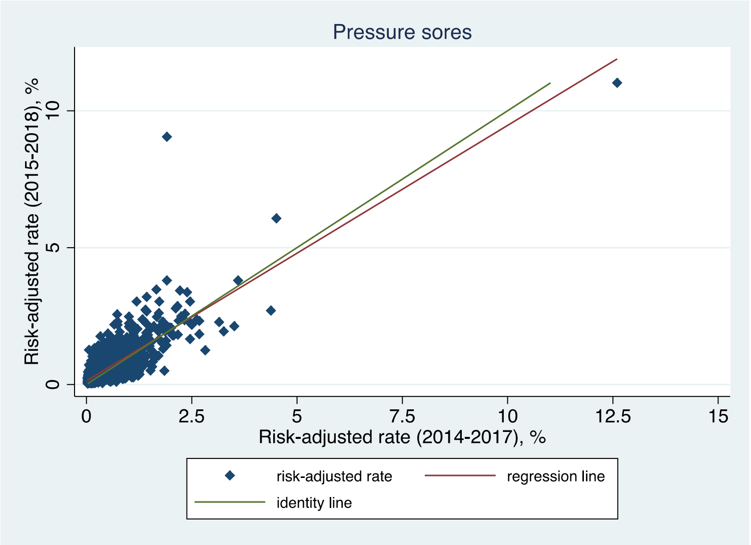

© 2021 Glance LG, et al. *JAMA Network Open.*

eFigure 2.23

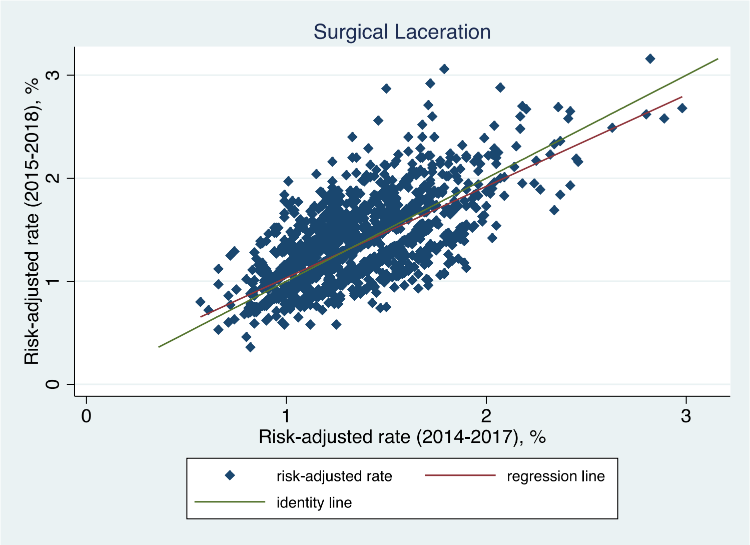

eFigure 2.24

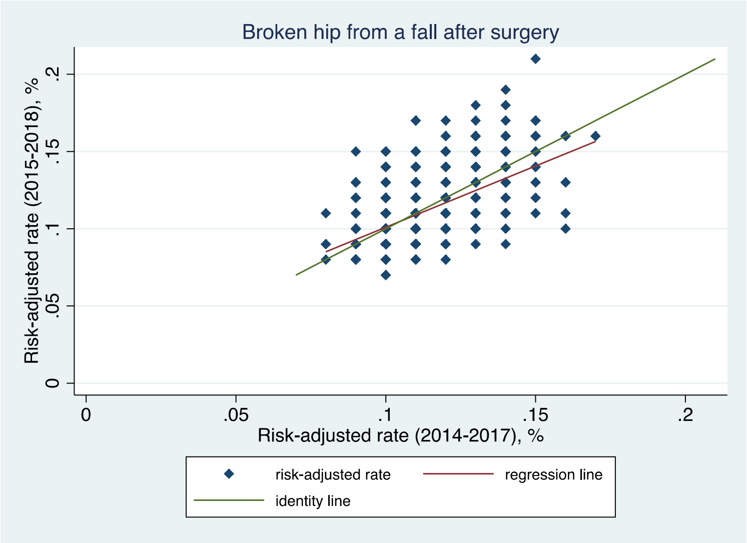

© 2021 Glance LG, et al. *JAMA Network Open.*

eFigure 2.25

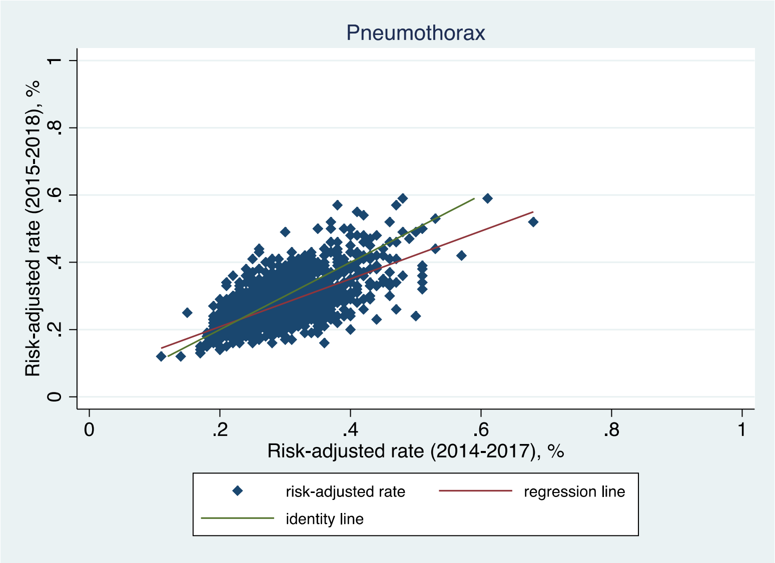

eFigure 2.26

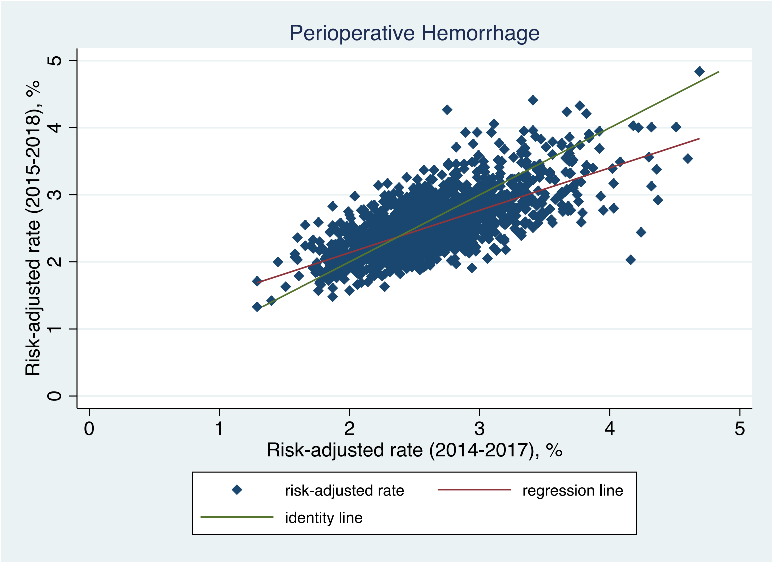

© 2021 Glance LG, et al. *JAMA Network Open.*
